# An Adaptive Fisher’s Combination Method for Joint Analysis of Multiple Phenotypes in Association Studies

**DOI:** 10.1038/srep34323

**Published:** 2016-10-03

**Authors:** Xiaoyu Liang, Zhenchuan Wang, Qiuying Sha, Shuanglin Zhang

**Affiliations:** 1 Department of Mathematical Sciences, Michigan Technological University, Houghton, Michigan, USA

## Abstract

Currently, the analyses of most genome-wide association studies (GWAS) have been performed on a single phenotype. There is increasing evidence showing that pleiotropy is a widespread phenomenon in complex diseases. Therefore, using only one single phenotype may lose statistical power to identify the underlying genetic mechanism. There is an increasing need to develop and apply powerful statistical tests to detect association between multiple phenotypes and a genetic variant. In this paper, we develop an Adaptive Fisher’s Combination (AFC) method for joint analysis of multiple phenotypes in association studies. The AFC method combines p-values obtained in standard univariate GWAS by using the optimal number of p-values which is determined by the data. We perform extensive simulations to evaluate the performance of the AFC method and compare the power of our method with the powers of TATES, Tippett’s method, Fisher’s combination test, MANOVA, MultiPhen, and SUMSCORE. Our simulation studies show that the proposed method has correct type I error rates and is either the most powerful test or comparable with the most powerful test. Finally, we illustrate our proposed methodology by analyzing whole-genome genotyping data from a lung function study.

To date, genome-wide association studies (GWAS) have become a tool of choice for the identification of genetic variants associated with complex human diseases. Currently, the analyses of most GWAS have been performed on a single phenotype. There is increasing evidence showing that pleiotropy, the effect of one variant on multiple phenotypes, is a widespread phenomenon in complex diseases^1^,^2^. Therefore, using only one single phenotype may lose statistical power to identify the underlying genetic mechanism. By taking into account the correlated structure of multiple phenotypes, we can not only discover genetic variants influencing multiple phenotypes that may lead to better understanding of etiology of complex human diseases^3^,^4^, but also can improve the statistical power by aggregating multiple weak effects and provide new biological insights by revealing pleiotropic variants^5^,^6^,^7^. Consequently, there is an increasing need to develop powerful statistical methods to detect association between multiple phenotypes and genetic variants.

Recently, several statistical methods for detecting association using multivariate phenotypes have been developed^8^,^9^,^10^,^11^,^12^,^13^. These methods can be divided into three groups: regression models, variable reduction method, and combining test statistics from univariate analysis^14^. Regression models, such as linear mixed effects models, generalized mixed effects models, and generalized estimating equations, can be used to test the association between a genetic variant and multiple phenotypes. By using random effects to account for correlation among individuals, linear and generalized mixed effect models can model the covariance structure not only caused by correlated phenotypes, but also caused by population structure^9^,^15^,^16^,^17^,^18^. Generalized estimating equations collapse random effects and random residual errors in the model^19^. Existing variable reduction methods can be roughly divided into three categories, principal components analysis of phenotypes (PCP)^20^, canonical correlation analysis (CCA)^10^ and principal component of heritability (PCH)^11^,^21^. The PCP approach applies a dimension reduction technique and tests for associations between genetic variants and the principle components of the phenotypes rather than the individual phenotypes. CCA provides a convenient statistical framework to simultaneously test the association between any number of quantitative phenotypes and any number of genetic variants genotyped across a gene or region of interest for unrelated individuals. For each genetic variant, the PCH approach reduces the phenotypes to a linear combination of phenotypes that has the highest heritability among all linear combinations of the phenotypes. Based on PCH, several advanced methods have been proposed such as penalized PCH applicable to high-dimensional data^22^,^23^ and principle components of heritability with coefficients maximizing the quantitative phenotype locus heritability (PCQH)^11^,^24^,^25^. The third group, combining test statistics from univariate tests, is to conduct univariate analysis on each phenotype, then combine the univariate test statistics^26^. The Trait-based Association Test that uses Extended Simes procedure (TATES)^12^ belongs to this group. TATES combines p-values obtained in standard univariate GWAS while correcting for the correlation between p-values.

Motivated by TATES, in this article, we propose an Adaptive Fisher’s Combination (AFC) method for joint analysis of multiple phenotypes in genetic association studies. We first test the association between each of the phenotypes and a genetic variant of interest using standard GWAS software. Then, AFC uses the optimal number of p-values which is determined by the data to test the association. Using extensive simulation studies, we evaluate the performance of the proposed method and compare the power of the proposed method with the powers of TATES, Tippett’s method^27^, Fisher’s Combination test (FC)^28^, Multivariate Analysis of Variance (MANOVA)^29^, joint model of Multiple Phenotypes (MultiPhen)^8^, and Sum Score method (SUMSCORE)^12^. Our simulation studies show that the proposed method has correct type I error rates and is either the most powerful test or comparable with the most powerful tests. Finally, we illustrate our proposed methodology by analyzing whole-genome genotyping data from a lung function study.

## Method

Consider a sample of *n* unrelated individuals. Each individual has *K* phenotypes. Denote *Y*_*k*_ = (*y*_1*k*_, …, *y*_*nk*_)^*T*^ as the *k*^*th*^ phenotype of *n* individuals. Denote *X* = (*x*_1_, …, *x*_*n*_)^*T*^ as the genotypic score of *n* individuals at a genetic variant of interest, where *x*_*i*_ ∈ {0, 1, 2} is the number of minor alleles that the *i*^*th*^ individual carries at the genetic variant. We propose a new method to test the null hypothesis *H*_0_: none of the *K* phenotypes are associated with the genetic variant.

We test the association between each phenotypic vector *Y*_*k*_ (*k* = 1, 2, …, *K*) and the genotypic score *X* using a standard GWAS software (e.g. PLINK, Gen/ProbABEL, MaCH, SNPTEST, and FaST-LMM)^30^,^31^,^32^,^33^,^34^,^35^,^36^. Let *p*_1_, *p*_2_, …, *p*_*K*_ denote the p-values obtained by the standard univariate GWAS. Based on these p-values, we propose an Adaptive Fisher’s Combination (AFC) method to test the association between multiple phenotypes and the genetic variant. Let *p*_(*k*)_ denote the *k*^*th*^ smallest p-value, 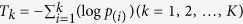, and 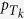 denote the p-value of *T*_*k*_. The statistic of AFC to test the association between the K phenotypes and the genetic variant is given by 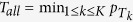. We use the following permutation procedure to evaluate the p-values of *T*_*k*_ and *T*_*all*_.

1. In each permutation, we randomly shuffle the genotypes and recalculate *p*_(1)_, …, *p*_(*K*)_ and *T*_1_, …, *T*_*K*_. Suppose that we perform *B*. times of permutations. Let 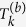 (*b* = 0, 1, …, *B*) denote the value of *T*_*k*_ based on the *b*^*th*^. permuted data, where *b* = 0 represents the original data.

2. We transfer 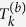 to 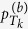 by


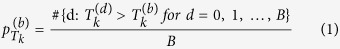


3. Let 
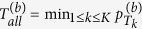
. Then, the p-vae of *T*_*all*_ is given by


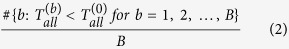


As shown in Appendix, the null distributions of *p*_1_, *p*_2_, …, *p*_*K*_ and thus of *T*_*all*_ do not depend on the genetic variant being tested. Thus, the permutation procedure described above to generate an empirical null distribution of *T*_*all*_ needs to be done only once for a GWAS.

The R code of AFC is available at Shuanglin Zhang’s homepage http://www.math.mtu.edu/~shuzhang/software.html.

### Comparison of Methods

We compare the performance of our method with those of TATES^12^, Tippett’s method^27^, Fisher’s Combination test (FC)^28^, Multivariate Analysis of Variance (MANOVA)^29^, joint model of Multiple Phenotypes (MultiPhen)^8^, and Sum Score method (SUMSCORE)^12^. Here we briefly introduce each of those methods using the notations in the method section.

#### TATES

Combine the *K* phenotype-specific p-values obtained in standard univariate GWAS to acquire one overall p-value, 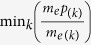, where *m*_*e*_ denotes the effective number of independent p-values of all *K* phenotypes, and *m*_*e*(*k*)_ denotes the effective number of p-values among the top *k* p-values.

#### MANOVA

Consider a multivariate multiple linear regression model: ***Y*** = *Xβ*^*T*^ + **ε**, where ***Y*** denotes the *n* × *K* matrix of phenotypes, *β*^*T*^ = (*β*_1_, …, *β*_*K*_) is a vector of coefficients corresponding to the *K* phenotypes, and **ε**. is the *n* × *K* matrix of random errors with each row of **ε** to be i.i.d. *MVN* (0, **Σ**), where **Σ** is the covariance matrix of **ε**. To test *H*_0_ : *β* = 0, the likelihood ratio test is equivalent to the Wilk’s Lambda test statistic of MANOVA^37^, that is, 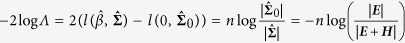. Here Λ denote the ratio of the likelihood function under *H*_0_ to the likelihood function under *H*_1_, *l*(*β*, ***Σ***) is the log-likelihood function, 
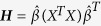
 and 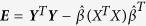, where 
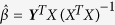
 is the maximum likelihood estimator (MLE) of *β*, and |·| denotes the determinant of a matrix. Then the test statistic has an asymptotic 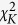 distribution^38^.

#### MultiPhen

By performing ordinaregression, MultiPhen develops a reversed analysis for joint analysis of multiple phenotypes by considering a genetic variant of interest *X* = (*x*_1_, …, *x*_*n*_)^*T*^ as a response variable, and the correlated phenotypes *Y*_*k*_ = (*y*_1*k*_, …, *y*_*nk*_)^*T*^ as predictors.

#### SUMSCORE

Let 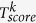 denote the score test statistic to test the association between the *k*^*th*^ phenotype and the genetic variant. The test statistic of SUMSCORE is given by 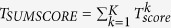. The p-value of *T*_*SUMSCORE*_ is estimated using a permutation procedure.

#### Tippett

The test statistic of Tippett is given by 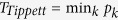. The p-value of *T*_*Tippett*_ is estimated using a permutation procedure.

#### FC

The Fisher’s combination test statistic is defined as 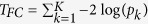. Yang *et al*.^28^ adopted three different approaches to calculate the p-value for correlated phenotypes. In this article, we calculate the p-value using a permutation procedure.

AFC, FC, and Tippett are closely related. Intuitively, when only one phenotype or very few phenotypes are associated with the variant, Tippett is more powerful than FC because in this case FC contains a lot of noises. When all phenotypes or a large proportion of the phenotypes are associated with the variant, FC is more powerful than Tippett because in this case Tippett only uses the minimum p-value and loses information. AFC can be adaptive to the number of phenotypes associated with the variant.

### Simulation

We generate genotype data at a genetic variant according to a minor allele frequency (MAF) under Hardy-Weinberg equilibrium. Phenotypes are generated similarly to that of van der Sluis *et al*.^12^. The phenotypic correlation structures mimic that of UK10K^39^, that is, the phenotypes are divided into several groups (factors) and the within-group correlation is larger than the between-group correlation. Denote *Y*_*k*_ = (*y*_1*k*_, …, *y*_*nk*_)^*T*^ as the *k*^*th*^ phenotype of *n* individuals and *X* = (*x*_1_, …, *x*_*n*_)^*T*^ as the genotypic score of the *n* individuals at the genetic variant.

#### Scenario 1

considering one factor model with genetic variant effect on the factor. We first generate a common factor, *f* = *βX*, where *f* is the *n* by 1 common factor and *β* is the effect size. Then we simulate *K* phenotypes by


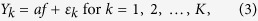


where *a* is a factor loading, *ε*_*k*_ = (*ε*_1*k*_, …, *ε*_*nk*_)^***T***^ ~ *MVN*(0, ***I***_***n***_), and ***I***_***n***_ is the identity matrix.

#### Scenario 2

considering 4 factor model with the genetic variant effect on the fourth factor, each factor has (*K*)/4 (*K* is a multiple of 4) phenotypes. We generate 4 correlated factors using (*f*_1_, *f*_2_, *f*_3_, *f*_4_)^***T***^ ~ *MVN*(0, **Σ**), where **Σ** = (1−*ρ*_*fa*_)***I*** + *ρ*_*fa*_ ***A***, ***A*** is a matrix with elements of 1, ***I*** is the identity matrix, and *ρ*_*fa*_ is the correlation between any two factors. Then, we transform the fourth factor *f*_4_ to 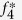
by 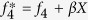 and simulate *K* phenotypes using


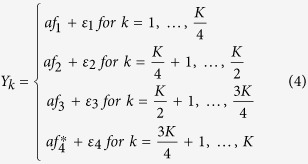


where *a* is a factor loading, *ε*_*j*_ = (*ε*_1*j*_, …, *ε*_*nj*_)^***T***^ ~ *MVN*(0, ***I***_***n***_) for *j* = 1, …, 4, and *β* is the effect size.

#### Scenario 3

considering two factor model with the genetic variant effect on the second factor, each factor has (*K*)/(2) (*K* is a multiple of 2) phenotypes. We generate two correlated factors using (*f*_1_, *f*_2_)^***T***^ ~ *MVN*(0, **Σ**), where **Σ** = (1−*ρ*_*fa*_)***I*** + *ρ*_*fa*_***A***, ***A*** is a matrix with elements of 1, ***I*** is the identity matrix, and *ρ*_*fa*_ is the correlation between any two factors. Then, we transform the second factor *f*_2_ to 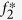
by 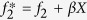 and simulate *K* phenotypes using


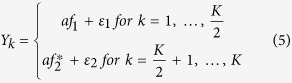


where *a* is a factor loading, *ε*_*j*_ = (*ε*_1*j*_, …, *ε*_*nj*_)^***T***^ ~ *MVN*(0, ***I***_***n***_) for *j* = 1, 2, and *β* is the effect size.

#### Scenario 4

considering 4 factor model with genetic variant effect specific to the *K*^*th*^ phenotype, each factor has (*K*)/4 (*K* is a multiple of 4) phenotypes. By using the original factors *f*_1_, *f*_2_, *f*_3_, *f*_4_ in scenario 2, we simulate *K* phenotypes using


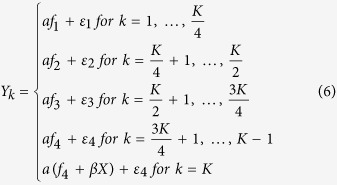


where *a* is a factor loading, *ε*_*j*_ = (*ε*_1*j*_, …, *ε*_*nj*_)^***T***^ ~ *MVN*(0, ***I***_***n***_) for *j* = 1, …, 4, and *β* is the effect size.

#### Scenario 5

considering one factor model with the genetic variant effect specific to the *K*^*th*^ phenotype. We simulate *K* phenotypes by


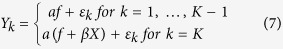


where *f*~*MVN*(0, ***I***_***n***_), *a* is a factor loading, *ε*_*k*_ = (*ε*_1*k*_, …, *ε*_*nk*_)^***T***^ ~ *MVN*(0, ***I***_***n***_), and *β* is the effect size.

#### Scenario 6

considering a network model, where the *K* phenotypes are correlated and the correlation structure is not due to one or multiple underlying common factors. We generate *K* phenotypes independent of genotypes for each individual by using 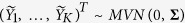, where **Σ** = (1 − *ρ*_*phe*_)***I*** + *ρ*_*phe*_***A***, ***A*** is a matrix with elements of 1, ***I*** is the identity matrix, and *ρ*_*phe*_ is the correlation between any two phenotypes. After generating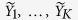, let 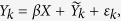 where *ε*_*k*_ = (*ε*_1*k*_, …, *ε*_*nk*_)^***T***^ ~ *MVN*(0, ***I***_***n***_).

In scenarios 2–5, the within-factor correlation is *a*^2^ and between-factor correlaiton is *a*^2^*ρ*_*fa*_. To evaluate type I error of the proposed method, we generate phenotypic values independent of genotypes by assigning *β* = 0. To evaluate power, we generate phenotypic values according to the six scenarios described above.

### Simulation results

We use two sets of simulations to evaluate the type I error rates of the proposed method. The first set of simulations is normal simulation studies and includes 10,000 replicated samples for each sample size under each scenario. The p-values are estimated using 10,000 permutations. For 10,000 replicated samples, the 95% confidence intervals (CIs) for type I error rates at the nominal levels 0.01 and 0.001 are (0.008, 0.012) and (0.0004, 0.0016), respectively. The estimated type I error rates of the proposed test (AFC) are summarized in Table 1. From Table 1, we can see that most of the estimated type I error rates are within the 95% CIs and those type I error rates not within the 95% CIs are very close to the bound of the corresponding 95% CI, which indicates that the proposed method is valid.

The second set of simulations mimics GWAS. To be comparable with the real data analysis, we generate 6,000 unrelated individuals with 8 phenotypes at 10^6^ variant sites under each scenario. The phenotypes are independent of genotypes. The MAF at each variant is a random number between 0.05 and 0.5. The null distributions of *T*_1_, …, *T*_*K*_ and *T*_*all*_ are generated by 10^6^ permutations using the genotypes at the first variant. We consider genotypes at 10^6^ variants as 10^6^ replicated samples. For 10^6^ replicated samples, the 95% confidence intervals (CIs) for the type I error rates at the nominal levels 10^−3^, 10^−4^, and 10^−5^ are (0.94×10^−3^, 1.06×10^−3^), (0.80 × 10^−4^, 1.20 × 10^−4^) and (0.38 × 10^−5^, 1.62 × 10^−5^), respectively. The estimated type I error rates of the proposed test (AFC) are summarized in Table 2. From Table 2, we can see that all of the estimated type I error rates are within the 95% CIs, which indicates that the proposed method is valid.

**Figure 1:**
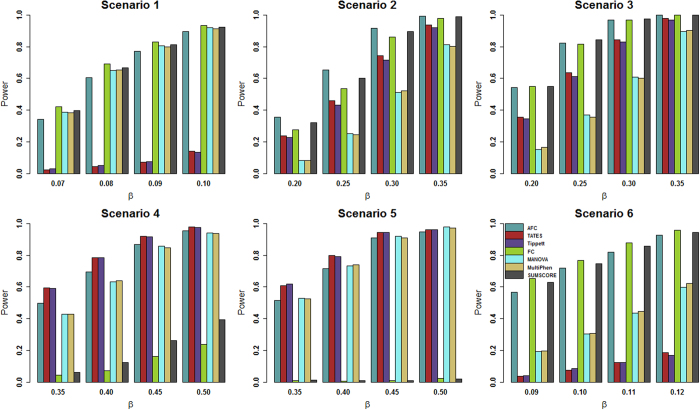
Power comparisons of the 7 tests for power as a function of effect size (*β*) under the 6 scenarios. The total number of phenotypes is 20. The sample size is 1,000. MAF is 0.3. The factor loadings are 0.75. In scenarios 2, 3 and 4, the factorial correlation (*ρ*_*fa*_) is 0.1. In scenario 6, the phenotypic correlation (*ρ*_*phe*_) is 0.1. The powers are evaluated at 0.1% significance level.

**Figure 2:**
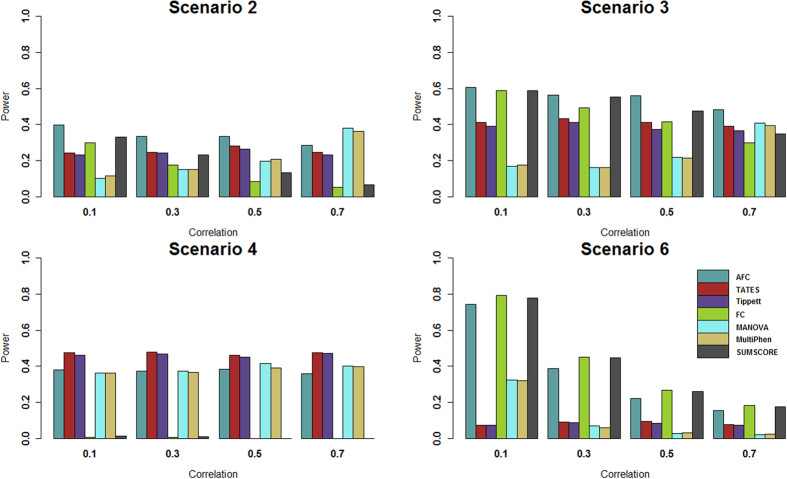
Power comparisons of the 7 tests for power as a function of factorial correlation (*ρ*_*fa*_) under scenarios 2 to 4, and as a function of phenotypic correlation (*ρ*_*phe*_) under scenario 6. The total number of phenotypes is 20. The sample size is 1,000. MAF is 0.3. The factor loadings are 0.75. In scenarios 2 and 3, the effect size (*β*) is 0.2. In scenario 4, the effect size (*β*) is 0.3. In scenario 6, the effect size (*β*) is 0.1. The powers are evaluated at 0.1% significance level.

**Figure 3:**
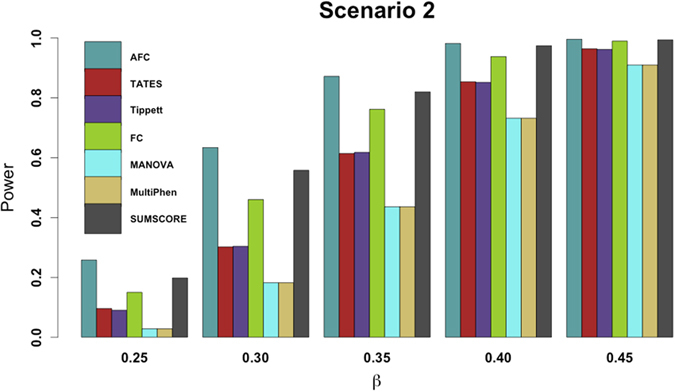
Power comparisons of the 7 tests for power as a function of effect size (*β*) under scenario 2. The total number of phenotypes is 20. The sample size is 1,000. MAF is 0.3. The factor loadings are 0.75. The factorial correlation (*ρ*_*fa*_) is 0.1. The powers are evaluated at 10-6 significance level while p-values of AFC, FC, Tippet, and SUMSCORE are evaluated by 107 permutations.

For power comparisons, we consider (1) power as a function of the effect size under all six scenarios, and (2) power as a function of factorial correlation (*ρ*_*fa*_) under scenarios 2–4 and power as a function of phenotypic correlation (*ρ*_*phe*_) under scenario 6 because scenarios 1 and 5 are one factor model and thus have no *ρ*_*fa*_ and *ρ*_*phe*_ involved. For Figs 1 and 2, the p-values of AFC, FC, Tippett, and SUMSCORE are estimated using 10,000 permutations and the p-values of TATES, MultiPhen, and MANOVA are estimated using asymptotic distributions. The powers of all tests are evaluated using 1,000 replicated samples at 0.1% significance level. For Fig. 3, the p-values of AFC, FC, Tippett, and SUMSCORE are estimated using 10^7^ permutations. The powers of all tests are evaluated using 1,000 replicated samples at 10^−6^ significance level.

Figure 1 gives the power comparisons of the 7 tests (AFC, TATES, Tippett, FC, MANOVA, MultiPhen, and SUMSCORE) for the power as a function of the effect size based on the six scenarios for 20 phenotypes. This figure shows that (1) AFC is either the most powerful test (genotypes directly impact on a portion of the phenotypes: scenarios 2–3) or comparable to the most powerful test (genotypes directly impact on all phenotypes or a single phenotype: scenarios 1, 4, 5, and 6); (2) TATES and Tippett have similar power and are much less powerful than other methods when genotypes directly impact on all phenotypes (scenarios 1 and 6); (3) MANOVA and MultiPhen have similar power and are much less powerful than other methods when genotypes directly impact on a portion of the phenotypes (scenarios 2–3); and (4) SUMSCORE and FC have similar power and are much less powerful than other methods when genotypes directly impact on a single phenotype (scenarios 4–5).

Power comparisons of the 7 tests for the power as a function of the factorial correlation (*ρ*_*fa*_) under scenarios 2–4 and as a function of the phenotypic correlation (*ρ*_*phe*_) under scenario 6 are given by Fig. 2. This figure shows that under scenario 4, the powers of all tests do not change with the factorial correlation because only one phenotype is associated with the variant and thus the factorial correlation does not change the information of association between the variant and phenotypes. Under scenarios 2, 3 and 6, (1) the powers of SUMSCORE and FC decrease with the increasing of the factorial or phenotypic correlation because SUMSCORE and FC involve all phenotypes and thus information contained by all phenotypes will decrease with the increasing of the factorial or phenotypic correlation; (2) the powers of TATES and Tippett do not change with the increasing of the factorial or phenotypic correlation because TATES and Tippett essentially only depend on the phenotype that has the strongest association with the variant; (3) under scenario 6, the power of AFC decreases with the increasing of the phenotypic correlation; under scenarios 2–3, the power of AFC does not change much with the factorial correlation; and (4) under scenario 6, the powers of MANOVA and MultiPhen decrease with the increasing of the phenotypic correlation; under scenarios 2–3, the powers of MANOVA and MultiPhen increase with the increasing of the factorial correlation, which is consistent with the results of Ray *et al*.^38^. We also give power comparisons of the 7 tests using a significance level of 10^−6^ with 10^7^ permutations and 1,000 replicates for the power as a function of effect size (β) under scenario 2 (Fig. 3). Figure 3 shows that the patterns of the power comparisons using significance level 10^−6^ are similar to that using a significance level of 0.1% in Fig. 1 (scenario 2).

In summary, the proposed method has correct type I error rates and is either the most powerful test or comparable with the most powerful tests. No other methods have consistently good performance under the six simulation scenarios.

### Application to the COPDGene

Chronic obstructive pulmonary disease (COPD) is one of the most common lung diseases characterized by long term poor airflow and is a major public health problem^40^. The COPDGene Study^41^ (http://www.ncbi.nlm.nih.gov/projects/gap/cgi-bin/study.cgi?study_id=phs000179.v1.p1) is a multi-center genetic and epidemiologic investigation to study COPD. This study is sufficiently large and appropriately designed for genome-wide association analysis of COPD. In this study, a total of more than 10,000 subjects have been recruited including non-Hispanic Whites (NHW) and African-Americans (AA). The participants in this study have completed a detailed protocol, including questionnaires, pre- and post-bronchodilator spirometry, high-resolution CT scanning of the chest, exercise capacity (assessed by six-minute walk distance), and blood samples for genotyping. The participants were genotyped using the Illumina OmniExpress platform. The genotype data have gone through standard quality-control procedures for genome-wide association analysis detailed at http://www.copdgene.org/sites/default/files/GWAS_QC_Methodology_20121115.pdf. Variants with MAF <1% were excluded in the data set.

**Figure 4:**
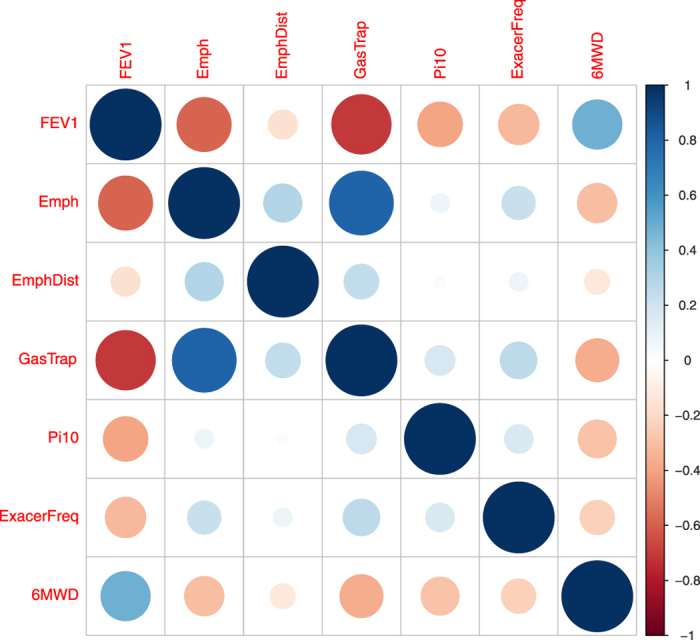
The correlation matrix plot of the 7 COPD-related phenotypes.

To evaluate the performance of our proposed method on a real data set, we applied all of the 7 methods to the COPDGene of NHW population to carry out GWAS of COPD-related phenotypes. Based on the literature studies of COPD^42^,^43^, we selected 7 key quantitative COPD-related phenotypes, including FEV1 (% predicted FEV1), Emphysema (Emph), Emphysema Distribution (EmphDist), Gas Trapping (GasTrap), Airway Wall Area (Pi10), Exacerbation frequency (ExacerFreq), Six-minute walk distance (6MWD), and 4 covariates, including BMI, Age, Pack-Years (PackYear) and Sex. EmphDist is the ratio of emphysema at −950 HU in the upper 1/3 of lung fields compared to the lower 1/3 of lung fields. Followed by Chu *et al*.^42^, we did a log transformation on EmphDist in the following analysis. The correlation structure of the 7 COPD-related phenotypes is given in Fig. 4. In the analysis, participants with missing data in any of the 11 variables were excluded. Therefore, a complete set of 5,430 individuals across 630,860 SNPs were used in the following analyses. In the analysis, we first adjusted each of the 7 phenotypes for the 4 covariates using linear models. Then, we performed the analysis based on the adjusted phenotypes.

To identify SNPs associated with the phenotypes, we adopted the commonly used genome-wide significance level 5 × 10^−8^. The results were summarized in Table 3. There were total 14 SNPs in Table 3. All of the 14 SNPs had previously been reported to be in association with COPD by eligible studies^44^,^45^,^46^,^47^,^48^,^49^,^50^,^51^,^52^,^53^,^54^,^55^,^56^,^57^. From Table 3, we can see that MultiPhen identified 14 SNPs; MANOVA identified 13 SNPs; AFC identified 12 SNPs, FC and SUMSCORE identified 10 SNPs; and TATES and Tippett identified 9 SNPs. Among the five methods based on combining test statistics from univariate analysis (AFC, TATES, Tippett, FC, and SUMSCORE), AFC identified 2 or 3 more genome-wide significant SNPs than the other 4 methods.

## Discussion

GWAS have identified many variants with each variant affecting multiple phenotypes, which suggests that pleiotropic effects on human complex phenotypes may be widespread. Therefore, statistical methods that can jointly analyze multiple phenotypes in GWAS may have advantages over analyzing each phenotype individually. In this article, we developed a new method AFC to jointly analyze multivariate phenotypes in genetic association studies. We used extensive simulation studies as well as real data application to compare the performance of AFC with TATES, Tippett, FC, MANOVA, MultiPhen, and SUMSCORE. Our simulation results showed that AFC has correct type I error rates. With respect to power, AFC is either the most powerful test or has similar power with the most powerful test under a variety of simulation scenarios. Additionally, the real data analysis results demonstrated that the proposed method has great potential in GWAS on complex diseases with multiple phenotypes such as COPD.

AFC has several important advantages. First, it allows researchers to test genetic associations using standard GWAS software. Second, phenotypes of different types (e.g., dichotomous, ordinal, continuous) can be easily analyzed simultaneously. Third, since AFC is based on p-values obtained from standard univariate GWAS, it can not only test the association between multiple phenotypes and one genetic variant of interest, but also can test the association between multiple phenotypes and multiple genetic variants. For common variants, multiple-variant AFC can be applied based on p-values obtained in standard univariate GWAS for each variant and each phenotype. For rare variants, we can first combine genotypes of rare variants by giving different weights, hoping that we give big weights to the variants having strong associations with the phenotypes. Then, we can apply AFC to test the association between the combined genotypes and multiple phenotypes. In conclusion, we showed that our proposed method provides a useful framework for joint analysis of multiple phenotypes in association studies.

It is well known that the effect sizes of identified variants are often small and that a large sample size is necessary to ensure sufficient power to detect such variants. A common strategy to increase sample size is to perform a meta-analysis by combining summary statistics from a series of studies. The proposed AFC method can be applied to meta-analysis. Suppose that there are *L* independent studies containing the variant of interest and each study has *K* phenotypes. Let *T*_1*l*_, …, *T*_*Kl*_ denote the summary statistics from the *l*^*th*^ study. Suppose that *T*_*l*_ = (*T*_1*l*_, …, *T*_*Kl*_)^*T*^~*N*(0,Σ_*l*_) under the null hypothesis, where Σ_*l*_ can be estimated from the values of *T*_*l*_ for all independent SNPs in the GWAS from the *l*^*th*^ study^58^. Then, 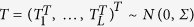, where Σ = *diag* (Σ_1_, …, Σ_*L*_). From *T*, we can calculate the corresponding p-values 
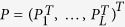
, where *P*_*l*_ = (*p*_1*l*_, …, *p*_*Kl*_)^*T*^. The AFC test statistic is based on the p-values *P*. In the permutation procedure, we can generate *T* according to the distribution *N*(0,Σ) and then we can calculate the p-values *P* in each permutation.

## Additional Information

**How to cite this article**: Liang, X. *et al*. An Adaptive Fisher’s Combination Method for Joint Analysis of Multiple Phenotypes in Association Studies. *Sci. Rep.* **6**, 34323; doi: 10.1038/srep34323 (2016).

## Supplementary Material

Supplementary Information

## Figures and Tables

**Table 1:** The estimated type I error rates of the proposed method for MAF equals 0.3.

*α*	Sample size	Scenario					
		1	2	3	4	5	6
0.01	1000	0.0088	0.0110	0.0105	0.0083	0.0083	0.0108
	2000	0.0095	0.0107	0.0094	0.0083	0.0098	0.0110
0.001	1000	0.0008	0.0015	0.0012	0.0008	0.0007	0.0012
	2000	0.0015	0.0014	0.0007	0.0009	0.0011	0.0014

*α* is the significance level. 10,000 replicates are used in the simulations.

**Table 2:** The estimated type I error rates of the proposed method that mimic GWAS.

*α*	Scenario					
	1	2	3	4	5	6
1.00 × 10^−3^	1.02 × 10^−3^	1.06 × 10^−3^	0.94 × 10^−3^	1.03 × 10^−3^	1.00 × 10^−3^	1.05 × 10^−3^
1.00 × 10^−4^	1.03 × 10^−4^	1.20 × 10^−4^	0.80 × 10^−4^	0.97 × 10^−4^	1.20 × 10^−4^	0.82 × 10^−4^
1.00 × 10^−5^	1.30 × 10^−5^	1.10 × 10^−5^	1.50 × 10^−5^	1.40 × 10^−5^	1.00 × 10^−5^	0.50 × 10^−5^

*α* is the significance level.

**Table 3:** Significant SNPs and the corresponding p-values in the analysis of COPDGene.

Chr	Position	Variant identifier	AFC	TATES	Tippett	FC	MANOVA	MultiPhen	SUM-SCORE
4	145431497	rs1512282	1.10 × 10^−8^	5.77 × 10^−9^	8.00 × 10^−9^	6.00 × 10^−9^	1.69 × 10^−9^	1.03 × 10^−9^	2.00 × 10^−8^
4	145434744	rs1032297	0	6.22 × 10^−13^	0	0	6.52 × 10^−14^	7.69 × 10^−14^	0
4	145474473	rs1489759	0	2.52 × 10^−16^	0	0	1.11 × 10^−16^	1.22 × 10^−16^	0
4	145485738	rs1980057	0	9.35 × 10^−17^	0	0	6.68 × 10^−17^	8.14 × 10^−17^	0
4	145485915	rs7655625	0	1.64 × 10^−16^	0	0	7.12 × 10^−17^	9.13 × 10^−17^	0
15	78882925	rs16969968	0	2.98 × 10^−8^	4.90 × 10^−8^	1.00 × 10^−8^	1.32 × 10^−11^	7.84 × 10^−12^	3.33 × 10^−8^
15	78894339	rs1051730	0	2.63 × 10^−8^	4.20 × 10^−8^	9.00 × 10^−9^	1.41 × 10^−11^	8.16 × 10^−12^	1.00 × 10^−8^
15	78898723	rs12914385	0	5.14 × 10^−10^	0	0	1.76 × 10^−12^	1.48 × 10^−12^	0
15	78911181	rs8040868	0	2.40 × 10^−9^	5.00 × 10^−9^	0	2.74 × 10^−12^	2.59 × 10^−12^	0
15	78878541	rs951266	0	**5.17 × 10^−8^**	**8.10 × 10^−8^**	1.50 × 10^−8^	1.77 × 10^−11^	1.02 × 10^−11^	2.15 × 10^−8^
15	78806023	rs8034191	1.40 × 10^−8^	**1.02 × 10^−7^**	**1.70 × 10^−7^**	**9.50 × 10^−8^**	2.14 × 10^−10^	7.74 × 10^−11^	**8.43 × 10^−8^**
15	78851615	rs2036527	2.90 × 10^−8^	**1.56 × 10^−7^**	**2.41 × 10^−7^**	**1.12 × 10^−7^**	3.99 × 10^−10^	1.77 × 10^−10^	**2.01 × 10^−7^**
15	78826180	rs931794	**6.30 × 10^−8^**	**1.18 × 10^−7^**	**1.94 × 10^−7^**	**2.67 × 10^−7^**	2.35 × 10^−10^	9.09 × 10^−11^	**3.32 × 10^−7^**
15	78740964	rs2568494	**5.00 × 10^−6^**	**2.88 × 10^−5^**	**3.42 × 10^−5^**	**1.34 × 10^−5^**	**1.05 × 10^−7^**	4.23 × 10^−8^	**2.11 × 10^−5^**

The p-values of AFC, Tippett, FC, and SUMSCORE are evaluated using 10^9^ permutations. The p-values of TATES, MANOVA, and MultiPhen are evaluated using asymptotic distributions. The bold p-values indicate the p-values > 5 × 10^−8^.
