# Supplementary material for: An Adaptive Fisher’s Combination Method for Joint Analysis of Multiple Phenotypes in Association Studies
Source: Sci Rep. 2016 Oct 3;6:34323. doi: 10.1038/srep34323 (PMC5046106; doi:10.1038/srep34323)
Supplement: Supplementary Information [file srep34323-s1.doc]

**An Adaptive Fisher’s Combination Method for Joint Analysis of Multiple Phenotypes in Association Studies**

Xiaoyu Liang^1^, Zhenchuan Wang^1^, Qiuying Sha^1^, Shuanglin Zhang^1,*^

^1^Department of Mathematical Sciences, Michigan Technological University, Houghton, Michigan

**Appendix**

Without loss of generality, we assume that all phenotypes are quantitative. We use the linear model $y_{ij}=\beta_{0j}+\beta_{1j}x_{i}+\varepsilon_{i}$ to relate the $j^{th}$ phenotype and the genotype. Let $T_{j}$ denote the score test statistic to test the null hypothesis $H_{0}:\beta_{1j}=0$. Then, $T_{j}$ is given by

$T_{j}=U_{j}/\sqrt{V_{j}}$,

where $U_{j}=\sum_{i=1}^{n} y_{ij}(x_{i}-\bar{x})$ and $V_{j}=\sum_{i=1}^{n} (y_{ij}-\bar{y}_{j})^{2}\sum_{i=1}^{n} (x_{i}-\bar{x})^{2}/n$. Under the null hypothesis, the statistic $T_{j}$ [asymptotically](http://www.google.com/search?hl=zh-CN&lr=&sa=X&oi=spell&resnum=0&ct=result&cd=1&q=asymptotically&spell=1) follows a standard normal distribution. It is reasonable to assume that $T=\left(T_{1},\ldots,T_{K}\right)^{T}$ follows a multivariate normal distribution with mean 0 and covariance matrix $\Sigma$ under the null hypothesis^1^. Note that $\frac{1}{n}\sum_{i=1}^{n} (y_{ij}-\bar{y}_{j})^{2}\to\mathrm{var}\left(y_{1j}\right)=\sigma_{j}^{2}$, where $\sigma_{j}^{2}$ is the variance of the $j^{th}$ phenotype. We have

$$\begin{matrix} \mathrm{cov}\left(T_{l},T_{s}\right)\to\frac{E\left(\sum_{i=1}^{n} \sum_{j=1}^{n} \left(y_{il}-E\left(y_{1l}\right)\right)\left(y_{js}-E\left(y_{1s}\right)\right)\left(x_{i}-\bar{x}\right)\left(x_{j}-\bar{x}\right)\right)}{\sigma_{l}\sigma_{s}\sum_{i=1}^{n} \left(x_{i}-\bar{x}\right)^{2}} \\ =\frac{\mathrm{cov}\left(y_{1l},y_{1s}\right)\sum_{i=1}^{n} \left(x_{i}-\bar{x}\right)^{2}}{\sigma_{l}\sigma_{s}\sum_{i=1}^{n} \left(x_{i}-\bar{x}\right)^{2}}=\frac{\mathrm{cov}\left(y_{1l},y_{1s}\right)}{\sigma_{l}\sigma_{s}}=\rho_{ls} \end{matrix}$$

as $n\to\infty$, where $\rho_{ls}$ denotes the correlation coefficient between the $l^{th}$ phenotype and the $s^{th}$ phenotype. We can see that under null hypothesis, the distribution of $T=\left(T_{1},\ldots,T_{K}\right)^{T}$, and thus distributions of $p_{1},\ldots,p_{K}$ and $T_{all}$, are independent of genotypes.

Using the same arguments as above, we can show that if $p_{1},\ldots,p_{K}$ come from z score statistics or Wald statistics, distributions of $p_{1},\ldots,p_{K}$ and $T_{all}$ are also independent of the genotype^1^.

**References**

1. Zhu, X. *et al.* Meta-analysis of correlated traits via summary statistics from GWASs with an application in hypertension. *Am. J. Hum. Genet.* **96(1)**, 21-36 (2015).
